# Development of a specific stroke awareness tool for the Haitian community

**DOI:** 10.1371/journal.pgph.0005519

**Published:** 2025-12-11

**Authors:** Jude Hassan Charles, Leila Stewart, Emmanuel Gay, Selina Ancheta, Karelle Spence, Carolina M. Gutierrez, Valynncea Butler, Christina Ampie, Erika Marulanda, Nicole Sur, Negar Asdaghi, Hannah Gardener, Tatjana Rundek, Jose G. Romano, Gillian L. Gordon Perue

**Affiliations:** 1 Department of Neurology, Jackson Health System, Miami, Florida, United States of America; 2 Inter-Angency coordinator at United Nation High Commission for Refugee agency, Miami, Florida, United States of America; 3 Department of Neurology, Miller School of Medicine University of Miami, Miami, Florida, United States of America; New York University Grossman School of Medicine, UNITED STATES OF AMERICA

## Abstract

While stroke awareness tools are available in English and Spanish, none specifically serve the substantial Haitian population in the United States. To fill this gap, the Florida Stroke Registry (FSR) Education Core collaborated with Haitian Creole–speaking healthcare professionals and community members to create a stroke awareness tool tailored for the Haitian Creole community. This initiative led to the development of two candidate translations for the BE-FAST acronym (Balance, Eyes, Face, Arm, Speech, Time), a validated tool proven to improve patient outcomes by enabling earlier hospital presentation. An anonymous online survey was conducted across Florida, targeting healthcare professionals such as physicians, nurses, stroke coordinators, and rehabilitation specialists. Participants compared two proposed Haitian Creole translations of BE-FAST—FEL VIT and FE VIT—and evaluated their effectiveness and ability to communicate the urgency of seeking medical attention. Responses were analyzed using descriptive statistics, proportions with 95% confidence intervals, and chi-square testing for group differences. Among 46 survey respondents, 53% were women, and 41% were fluent in Haitian Creole. The professional breakdown was 56% physicians, 10% nurses, 20% stroke coordinators, and 13% rehabilitation specialists. Most participants (93%) agreed that a Haitian Creole stroke awareness tool would benefit their communities. FEL VIT (FIGI [face], EKILIB [equilibrium], LANGAJ [language/speech], VIZYON [vision/eye], IMOBILITE [immobility], TAN/TELEFON [time/call]) was selected as the preferred translation, with 46.7% favoring it over FE VIT (31.1%). FEL VIT was also rated highest for conveying the urgency needed for rapid hospital presentation following stroke symptoms (48.8% vs. 27.9%). Through collaboration across disciplines and cultures, the FSR successfully introduced FEL VIT, a Haitian Creole adaptation of BE-FAST, which was favored by stakeholders and is ready for implementation in community stroke awareness campaigns.

## Introduction

Scientific evidence demonstrates significantly better patients’ outcomes when acute stroke is identified and treated early [1]. The BE-FAST (Balance, Eyes, Face, Arm, Speech, Time) mnemonic is a recognized stroke awareness tool linked to earlier patient arrival times at hospital and better treatment outcomes as it facilitates expedited triage and time to treatments [2,3]. BE-FAST also has been demonstrated to have a higher sensitivity for identifying acute stroke appropriately in comparison to FAST (Face, Arm, Speech and Time) [3] including better performance in diverse populations, clinical setting and geographical regions such as rural vs urban setting [4]. Outside Haiti, Florida has one of the largest Haitian populations in the United States [5]. Within Florida there are known race-ethnic stroke disparities [6]. Unfortunately, studies comparing stroke among Haitians versus non-Haitians are limited, but a recent scoping review confirms evidence of gap in care for this population [7]. Current stroke awareness tools exist in English and Spanish, but none target the Haitian population.

## Aims and hypothesis

We hypothesize that it is feasible to create a Haitian Creole specific stroke awareness tool and that this tool would address this gap in community outreach and stroke awareness education among the Haitian community. To accomplish this the Florida Stroke Registry (FSR) collaborated with its Haitian stakeholders.

## Methods

### Ethics statement

The Florida Stroke Registry (FSR) is a statewide program focused on improving stroke care for all. The FSR received and maintains Institutional Review Board approval since its inception from the University of Miami Institutional Review Board (ID 20120987; CR00012124) [8,9]. It is a state funded program which utilizes data from 180 voluntarily participating stroke hospitals in Florida to drive quality improvement in stroke care across the state. As a recognized quality improvement program, the project ensured the anonymity of all participants; therefore, no formal consent was required or obtained.

### Setting and participants

The Florida Stroke Registry (FSR) Education Core, composed of four full-time members (CG, GGP, VB, and CA), led this project in collaboration with FSR stakeholders. Stakeholders are defined as members of the Florida Stroke Registry hospital community. A core workgroup was convened, initially including 10 members: stroke neurologists, an epidemiologist, stroke nurse educators, medical students, and a certified Haitian Creole translator (who volunteered specifically for this project but was not a typical FSR stakeholder). After development of the initial acronym versions, an additional Haitian-born stroke neurologist joined the group, bringing the total to 11 members.

### Development process

The project proceeded through the following steps (See S1 Fig):

1. Initial Request and Workgroup Formation: The FSR Education Core was approached by a Haitian-born stroke educator requesting a culturally appropriate tool for stroke awareness in Haitian Creole. Volunteers were solicited, and the workgroup was formed. The FSR education core created a small work group including a bilingual Haitian Creole FSR stakeholder and a certified Haitian Creole interpreter (EG) to translate the open access tool BE-FAST tool into Haitian Creole.
2. Translation and Drafting: A Haitian-speaking nurse educator led the initial line-by-line translation of the English acronym BEFAST into Haitian Creole. Drafts were developed in written form.
3. Iterative Review: The workgroup met twice monthly to review translations. Sessions focused on brainstorming appropriate terms, addressing grammatical issues, and considering oral comprehension, given that Haitian Creole is primarily a spoken language.
4. Consensus Process: All decisions regarding word choice and acronym structure required unanimous voting among the group. In cases of disagreement, the certified translator provided the deciding judgment.
5. Certified Translator Input: The translator reviewed all drafts, validating linguistic accuracy but also identifying challenges in producing direct equivalents for certain English phrases.
6. Stakeholder Review and Feedback: Draft acronyms, including the provisional FELVIT, were presented to the broader FSR stakeholder community. Feedback was obtained through a survey designed by the FSR Education Core, which was distributed to healthcare professionals working with Haitian patients.
7. Refinement and Expert Input: A Haitian-born stroke neurologist subsequently joined the project, shifting the group’s focus from literal translation toward meaning-based adaptation. This facilitated the transition to the finalized acronym FEL VIT, which allowed inclusion of additional stroke symptoms and optimized both accuracy and cultural resonance.
8. Approval: Final drafts were submitted to the FSR Executive leadership for formal approval.

The initial steps required over 2 months of work to identify a suitable translation. The first attempts at translation focused on the English template version and translating it letter-by-letter to Haitian Creole, any differences in opinion were referred to the Certified Creole interpreter. There were initial challenges in this direct 1:1 translation with key symptoms not accounted for by this method. The work group then met with a wider circle of native Haitian Creole speakers to identify possible replacements for these key missing terms in the new translation without success. The third iteration then focused on refinement and specifically expressing the idea of urgency or the message of BE-FAST not just to a phonetic translation. We sought to identify an appropriate phrase translation that would convey the urgency of stroke and the need to arrive immediately at the nearest stroke certified center. With this new focus, the new acronym underwent multiple iterations with feedback from the Florida Stroke Registry education core team to create two Haitian Creole iterations: FE VIT and FEL VIT. Both phrases urge the reader to act quickly with literal translations of FE VIT to English is “do it quick” in a general sense, while FEL VIT translated to English is “do this specific thing (call 911) very quick”. This small change gives the task in question more emphasis. In this case, it would imply going to the hospital as quickly as possible. The additional letter also allows FEL VIT to carry more details such as emphasizing facial droop and language problems.

**Fig 1:**
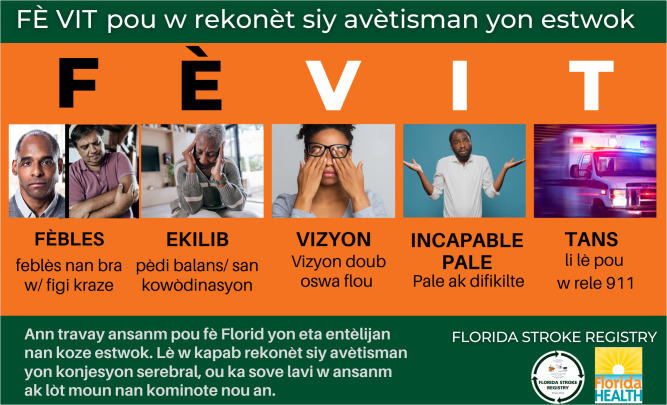
Pictorial representation of FE VIT for specific stroke awareness tool for the haitian community. Fig 1 represents the pictorial graph as presented to participants at the time of the survey. FE VIT is a Haitian creole phrase that means “be quick”. F for facial droop and arm weakness, E for loss of balance, V for loss of vision, I for incapable or difficulty speaking and T for time to call 911. Note should be made of 3 typographical errors: Fèbles (correct spelling is: Feblès), Incapable (the correct word should be enkapab as there is no “c” in Kreyol) and Tans (correct spelling is: Tan). These were not corrected for the manuscript to maintain historical accuracy. Fig 1 has since been improved to Fig 2, which shows is the preferred figure by the study participants and is the format recommended for final use or reproduction. Requests to use the final tool can be made at https//floridastrokecolloboration.org. Image Credit: University of Miami Miller School of Medicine.

The first sample FE VIT (see Fig 1) is described as follows: FEBLÉS (weakness), EKILIB (equilibrium), VIZYON (vision), ENKAPAB PALE (inability to speak) and Tan (time to call). Although FE VIT semantically conveys the level of understanding and a sense of urgency, it does not have the individualized key single words that carry the weight of stroke specific symptoms. Furthermore, the word ENKAPAB would be an attempt to use the French INCAPABLE, however this would not be a correct spelling in Haitian Creole.

**Fig 2:**
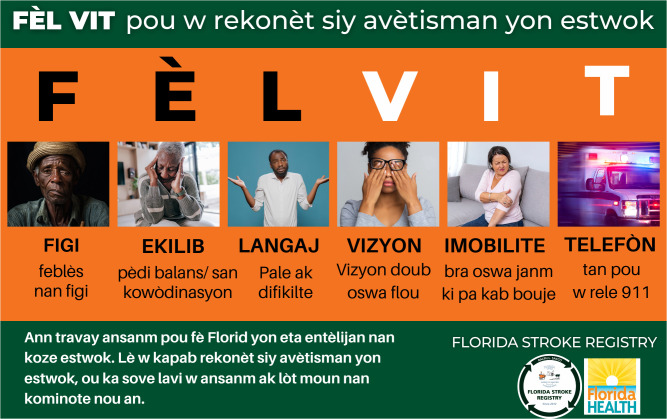
Pictorial representation of FEL VIT for Specific Stroke Awareness tool for the Haitian Community. FEL VIT is a Haitian creole phrase that means “be quick with a specific task”. F for facial droop, E for loss of balance, L language/difficulty speaking, V for loss of vision, I for arm weakness and T for time to call 911. Requests to use the final tool can be made at https//floridastrokecolloboration.org. Image Credit: University of Miami Miller School of Medicine.

The second sample FEL VIT see Fig 2 defined as follows: FIGI (face) EKILIB (equilibrium), LANGAJ (language/speech), VIZYON (vision/eye), IMOBILITE (paralysis/immobility) and TAN/TELEFON (time/call), incorporated the entirety of acronym in its meaning while still able to convey the same level of urgency.

These two final acronyms, FE VIT and FEL VIT were paired with appropriate images to explain their meaning. This educational content was subsequently formatted and printed for use in a public awareness campaign. All images presented were either developed from stock images or AI-generated specifically for the Florida Stroke Registry (FSR) using our Canva professional license. These images and this manuscript do not include any patient-related data. As such, no patient data or consent is required.

These final documents were presented to our FSR stakeholders and community partners fluent in Haitian Creole to determine the translation most suited for the Haitian Creole speaking community in Florida.

To address the second hypothesis question of suitability for our community, we designed a survey (See Table 1 for Survey questions) targeting mostly native Haitian Creole speaker and healthcare professionals with the prerequisite background knowledge of the intention of the tool. The survey was designed and built in Survey monkey platform. Our distribution strategy was to share the tool primarily within healthcare facilities which served large Haitian populations and to distribute the tool directly to local Haitian related medical societies. This tool was then distributed via email to these target individuals, FSR stakeholders, academic institutions and other community healthcare facilities located in South Florida between December 1^st^, 2023, and February 29^th^, 2024. As this is a quality improvement tool among medical professionals only, no consent was obtained and data was collected anonymously. Surveyed individuals were asked their opinion and preference among the two samples, and which one conveys best the clarity to recognize the stroke signs and symptoms and the urgency to seek medical assistance. The survey also inquired about the level of fluency in Haitian Creole, as well as an estimation of the Creole speaking patients they interact with in their community. Results were collected and analyzed using summary statistics to compare two proportions to determine which of the two tools would be most effective as a BE-FAST translations. Data was analyzed using Microsoft Excel (Version 2408 Build 1728.20114) to generate aggregated summary data and present results as percentages. Categorical variables were dichotomized and compared using chi-square tests to determine statistical associations. All reported p-values were two-sided and considered statistically significant at p < 0.05. Analyses were performed to evaluate differences in tool preference and survey responses, with appropriate methods selected based on variable type. Additional analysis was done to determine which sample would best convey the urgency of seeking medical attention to the Creole speaking population.

**Table 1:** Survey questions.

Q1. Is a Haitian Creole translation of B.E.F.A.S.T. for community outreach stroke awareness useful for your organization and/or in your community?
Q2. Can you describe or estimate the population size of Haitian Creole speakers in your community?
Q3. Aside from a Haitian Creole translation of B.E.F.A.S.T., is there another primary language(s) in Florida that the FSR should consider translating? Please indicate the language and where the population that speaks this language is located in Florida.
Q4. Are you fluent in Haitian Creole?
Q5. Please provide your feedback on SAMPLE 1 - FÈL VITFIGI - feblès nan figi face – facial weakness EKILIB - pèdi balans/ san kowòdinasyon equilibrium [balance] - loss of balance or coordination LANGAJ - difisil pou pale language – difficult to speak VIZYON - Vizyon doub oswa flou vision - double or blurred vision IMOBILITE - imobilite ak bra oswa janm immobility - immobility with arms or legs TELEFÒN - tan pou rele 911dial 911 - time to call 911FÈL VIT (Haitian Creole Be Fast)
Q6. Please provide your feedback on SAMPLE 2 - FÈ VITFÈBLES - feblès nan bra w/ figi krazeweakness - weakness in arms or face EKILIB - pèdi balans/ san Haitian Creole Stroke Awareness Design Feedback kowòdinasyon equilibrium [balance] - loss of balance or coordinationVIZYON - Vizyon doub oswa flouvision - double or blurred vision INCAPABLE PALE - pale ak difikilteincapable [French wording] of speaking - difficulty speakingTANS - li le pou w rele 911time - time to call 911FÈ VIT (Haitian Creole Be Fast)
Q7. Which of the two samples would you find most effective as a B.E.F.A.S.T. translation?
Q8. Which of the two sample designs best conveys the message of presenting to a hospital early/quickly once recognizing the signs or symptoms of stroke?

## Results

A total of 46/65 healthcare professionals participated in the survey which was distributed by convenience sampling providing a response rate of 70%. Among respondents, 93% agreed that translating the BE-FAST to Haitian Creole would be a useful and important tool to increase stroke awareness in their organizations and community. Most participants (88%) reported regular and frequent interaction with Haitian Creole speaking patients and 41% of participants were fluent in Haitian Creole. The survey was anonymous but 62% of participants opted to provide some demographic details; 56% including physicians, 53% women see Table 2. FEL VIT was identified as the most effective translation of BE-FAST 43% (95% CI: 32.9% – 60.9%) vs 31% (95% CI: 19.5% – 45.7%) for FE VIT (p = 0.37) and FEL VIT was thought to best convey the message of presenting to a hospital quickly once symptoms of stroke are recognized 48% (95% CI: 34.6% – 63.2%) vs. 28% (95% CI: 16.7% – 42.7%) (p = 0.035).

**Table 2:** Characteristics of healthcare professionals participating in the Haitian stroke awareness development tool survey (n = 30).

Characteristic	n	%
**Profession**		
Physicians	17	56%
Nurses	3	10%
Stroke Coordinators	6	20%
Rehabilitation Specialists	4	13%
**Gender**		
Women	16	53%
**Hospital Facility**		
Clinical Affiliation with Haitian Community (n = 46)	40	88%
**Language Fluency**		
Fluent in Haitian Creole (n = 46)	19	41%

This survey was anonymous, and only 60% of respondents provided limited demographic information. All participants were asked in question 4 their fluency in Haitian Creole.Percentages are based on available responses; totals may not equal 100% due to rounding and overlapping roles.

Slightly more than 40% of respondents took the time to provide written feedback on both translations. Feedback on FE VIT ranged from criticism, corrections, or negative feedback on the wording whereas none of the feedback for FEL VIT were negative (p = 0.0004). Survey respondents suggested other translations of BE-FAST would be helpful for their communities including Spanish translation 33%, French 10%, Portuguese 8%, Hindi 2%, Vietnamese 2%, and Tagalog 2%.

## Discussion

We demonstrate feasibility in creating a Haitian Creole specific stroke awareness tool, that can appropriately convey the urgency of stroke as well as to recognize and respond to a stroke. Overcoming this feasibility challenge is important as Haitian Creole is primarily an oral rather than written language, with most schools in Haiti employing French as the major language. The new FEL VIT tool also includes pictures as a visual aide to better convey the message.

Stroke care is highly time-sensitive, with numerous studies demonstrating that earlier intervention leads to better outcomes [1,10,11]. Since most strokes occur in the community rather than in inpatient settings, a focus on public knowledge of stroke awareness which allows for the rapid identification of stroke symptoms and prompt notification of emergency medical services by witnesses are crucial for appropriate and timely treatments [12]. The BEFAST tool has been shown to aide community witnesses in quickly identifying stroke symptoms [3,4]. Given its effectiveness in the general population, it is reasonable to assume that using such tools in a native language could reduce delays in hospital presentation for acute stroke patients. The literature supports and demonstrates that educating a community in its native language significantly increases stroke awareness and improves the community’s ability to identify and respond to stroke symptoms promptly [13].

To our knowledge, we report the first attempt at creating a Haitian specific stroke awareness tool. Such a tool has the potential to impact early recognition of stroke symptoms for Haitian patients both locally in Florida and internationally. In fact, in Haiti [7] the average stroke patient arrives more than 24 hours after their first stroke symptoms appear. FEL VIT is a very simple, easy to remember phrase in Haitian Creole. The concise format of FEL VIT allows it to be readily printable and reproducible on magnets, flyers or social media digital posts to improve dissemination within the community. Visit Florida Stroke Collaboration if you are interested in accessing the FEL VIT tool.

The FSR Education core selected the BEFAST tool as the model stroke awareness tool because BE FAST was developed and validated by reviewing a retrospective cohort with a final diagnosis of MRI confirmed stroke with evidence of improved performance in comparison with other stroke awareness tools [3]. The development process for FEL VIT used this validated tool as a foundation and then builds using linguistic and culturally appropriate themes with a specific focus on the target population.

Published literature suggests that strokes occur at an earlier age among the Haitians population compared to other ethnic groups, leading to early disability and a significant loss of quality of life [7]. This underscores the critical need for heightened stroke awareness within this population. Developing a tool in Haitian Creole is essential, as many Haitians living in South Florida do not speak or read English fluently. Studies have highlighted that language barriers are among the many challenges the Haitian population faces in accessing healthcare in the United States [14]. Our developed tool has been evaluated by native speakers and healthcare professionals who interact with Haitians daily. The use of a certified creole interpreter who is part of the Haitian diaspora is a particular strength of our project and the direct feedback from the Haitian Medical community selecting one of two options is an important part of community participatory research.

We have also demonstrated that both Creole speaking and non-Creole speaking healthcare professionals agree that a tool for this purpose is equally necessary and currently deficient, and that our provided translation of the FEL VIT tool is accurate and conveys the intended information with the needed level of urgency.

## Limitations

One limitation of this paper is the relatively small sample size, which may be due to the target group of the study comprising health care professionals knowledgeable about both stroke/the Stroke Awareness Tool and the Haitian community or those who speak Haitian Creole. To mitigate this limitation, we conducted a web-based survey to enhance access to respondents. There is a risk of sampling bias as participants were invited by convenience sampling to reach our target population. The sample size limits our statistical power so the results should be considered exploratory. Our Future study (FEL VIT part 2) has been designed and is currently undergoing FSR executive review. This second study which will focus on increasing stroke awareness among the Haitian community testing our newly developed FEL VIT tool against a control. This will include conducting a comparative analysis of the change in persons stroke awareness knowledge based on information presented with vs. without the FEL VIT tool using a pre and posttest along with 30 day recall of stroke symptoms. We aim to broaden the scope of our work by including both local, national and international Haitian communities using a web-based presentation of the FEL VIT tool. As it is focused on patients, we are aiming for a larger sample size of around 200 participants.

The rate of Haitian Creole fluency among study participants was 41%, which may be considered a limitation of the study. However, 88% of participants were healthcare professionals who regularly cared for Haitian Creole-speaking patients, and both percentages are higher than those typically observed in the general population of the United States.

Ideally the long-term impact would be to increase the number of patients from the Haitian community who recognize stroke symptoms and arrive within the acute stroke treatment window. We are however cognizant that this measure has been shown in published studies comparing Haitian vs Non-Haitians to be impacted by other factors such as insurance coverage and other non-clinical factors and is not only based on a patient’s knowledge about when to to seek care.[15].

## Conclusion

Through an interdisciplinary and diverse Florida Stroke Registry stakeholder membership, we have successfully translated the BE-FAST acronym into a Haitian Creole tool termed FEL VIT. The immediate impact of this work is the development of a tool Haitian Creole Stroke awareness tool that is reviewed and accepted by healthcare professionals within the Haitian community. FEL VIT can be used to increase stroke symptoms awareness among the Haitian population. We have also demonstrated that both Creole speaking and non-Creole speaking healthcare professionals agree that such a tool is equally necessary and efficient, and that the provided translation is accurate and conveys the intended information and urgency. We outline the process and encourage other medical societies or hospitals to consider translating the BE-FAST tools in the predominantly spoken language in their community to increase stroke awareness. Future directions will measure the impact of this tool on the Haitian Creole speaking community in Florida.

## Supporting information

S1 Fig Methodology of development of the Haitian specific creole tool. (PPTX)

S1 File STROBE checklist. (DOCX)
