# Supplementary figures and images for: Development of a specific stroke awareness tool for the Haitian community
Source: PLOS Glob Public Health. 2025 Dec 11;5(12):e0005519. doi: 10.1371/journal.pgph.0005519 (PMC12697954; doi:10.1371/journal.pgph.0005519)

## Slide 1
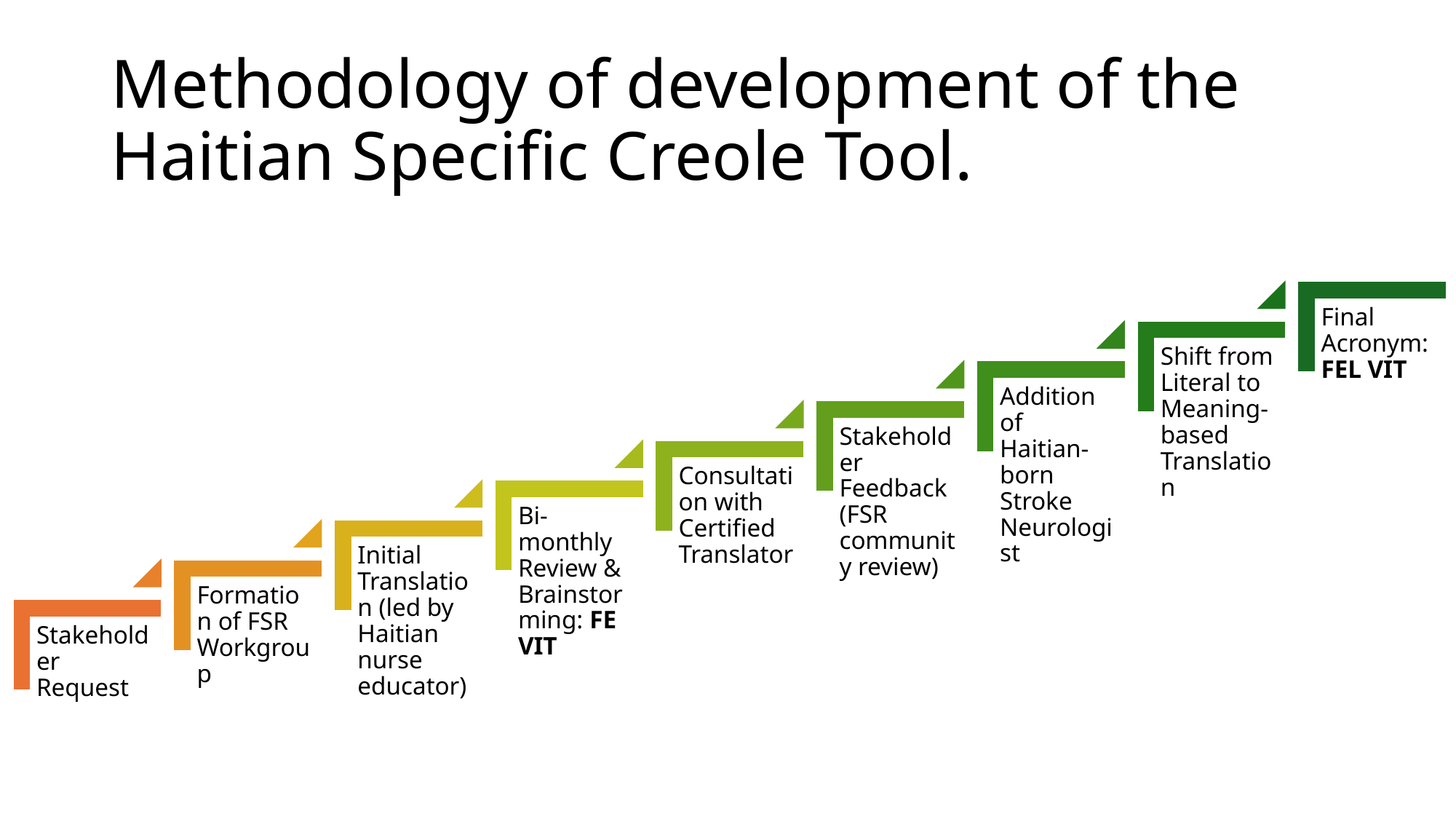

# Methodology of development of the Haitian Specific Creole Tool.

Supplement: S1 Fig — (PPTX) [file pgph.0005519.s001.pptx]
